# Supplementary material for: Nucleocapsid protein-specific monoclonal antibodies protect mice against Crimean-Congo hemorrhagic fever virus
Source: Nat Commun. 2024 Feb 26;15:1722. doi: 10.1038/s41467-024-46110-4 (PMC10897337; doi:10.1038/s41467-024-46110-4)
Supplement: Supplementary file 1 — Supplementary Information [file 41467_2024_46110_MOESM1_ESM.pdf]

Supplementary Materials for

Nucleocapsid protein-specific monoclonal antibodies protect mice against Crimean-Congo hemorrhagic fever virus

Aura R. Garrison[1,*], Vanessa Moresco[2], Xiankun Zeng[1], Curtis R. Cline[1], Michael Ward[1], Keersten Ricks[1], Scott Olschner[1], Lisa Cazares[1], Elif Karaaslan[3], Collin Fitzpatrick[1], Éric Bergeron[3], Scott D. Pegan[2,4] and Joseph W. Golden[1,*]

*Corresponding author. E-mail: joseph.w.golden.civ@health.mil or aura.r.garrison.civ@health.mil

This PDF file includes:
Figs. S1 to S5
Tables S1 to S3

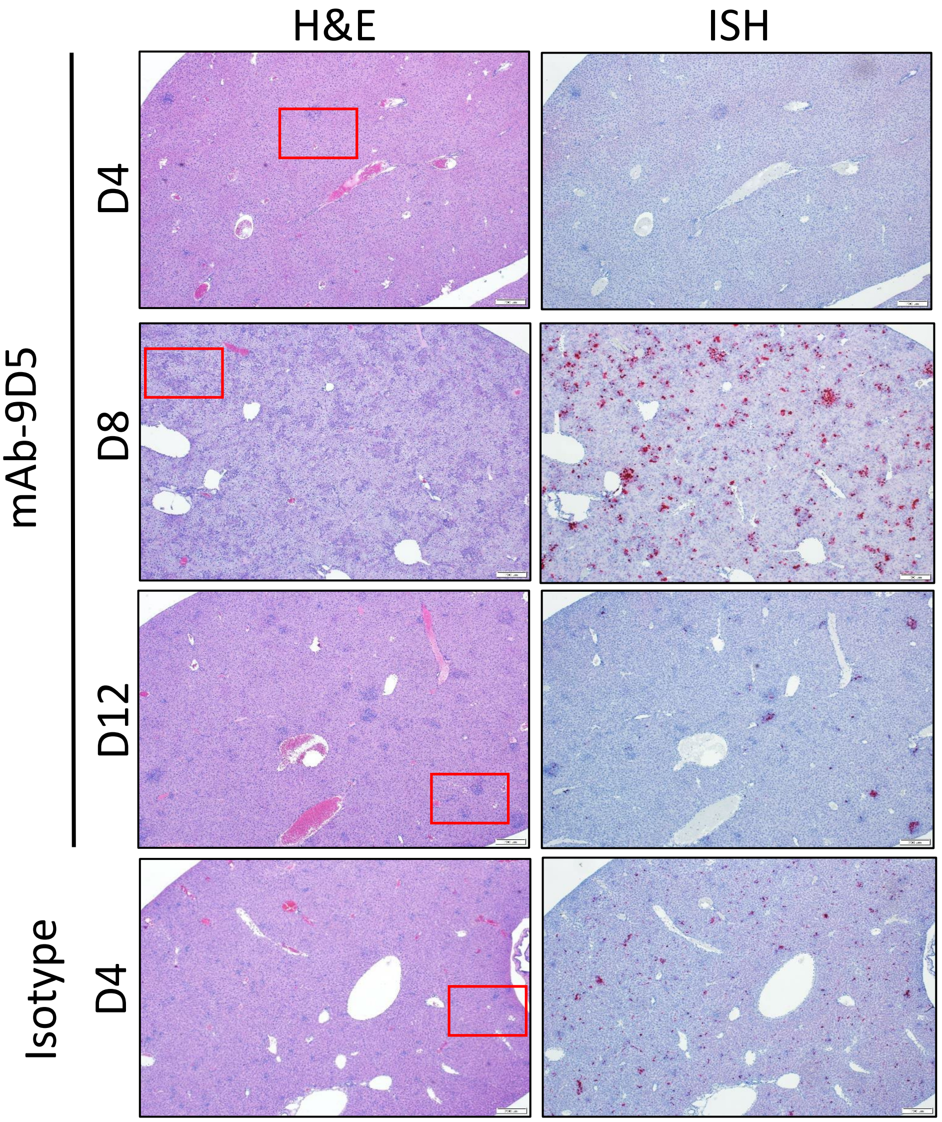

Figure S1. Histopathology and ISH of CCHFV infected mouse liver. Representative H&E ISH staining of livers of CCHFV strain IbAr 10200 infected mice treated with mAb-9D5 (days 4, 8 and 12) or isotype control antibody (day 4). Red box denotes the magnified regions shown in Figure 1B. Day 4 N=5 mice/group, day 8 N=5 mice mAb 9D5 treated, day 12 N=2 mAb 9D5 treated were evaluated and representative images depicting the most severe pathology are shown.

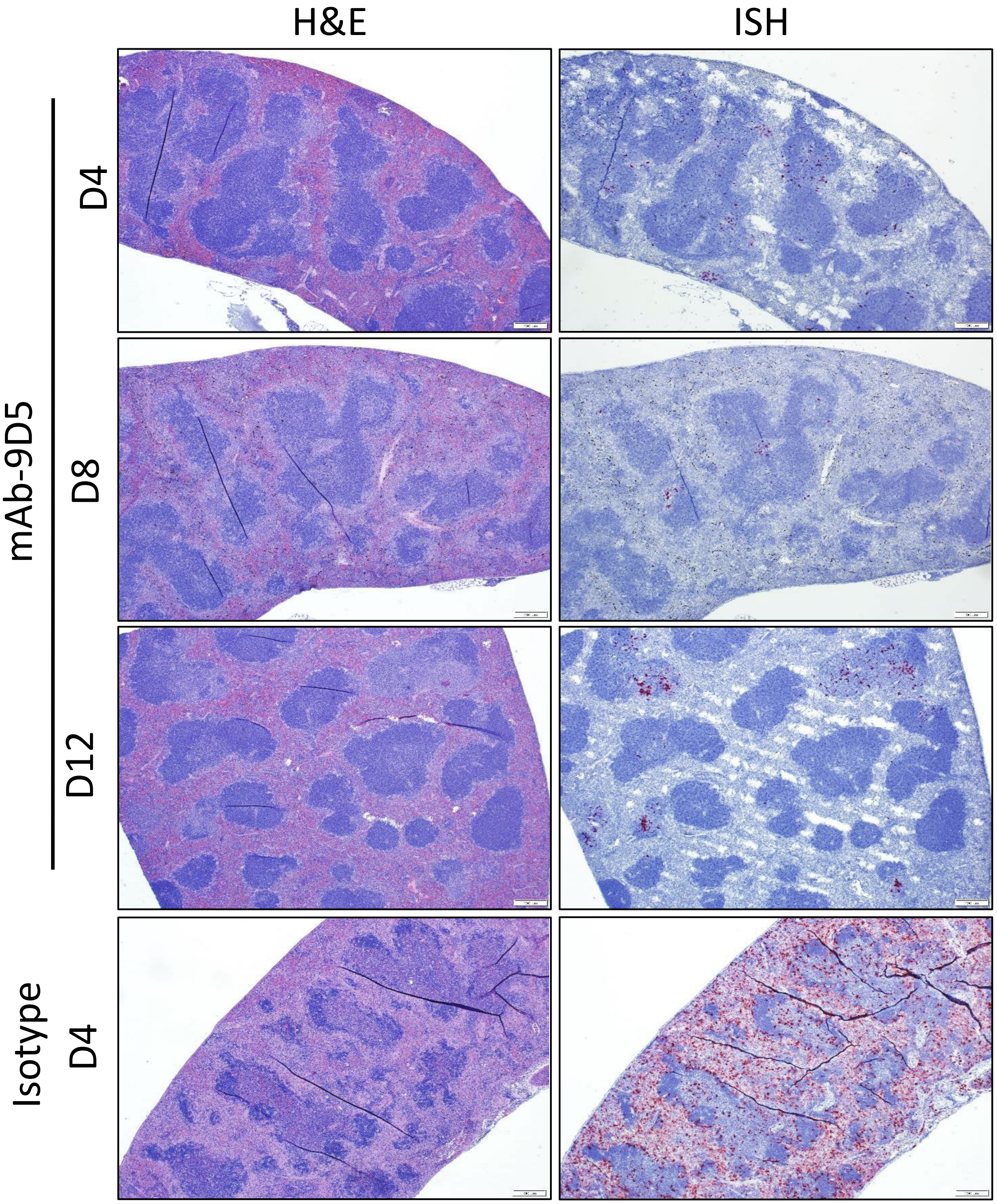

Figure S2. Histopathology and ISH of CCHFV infected mouse spleen. H&E and ISH comparison of spleen between mice treated with mAb-9D5 (days 4, 8 and 12) or isotype control animals (day 4 only) (4x magnification). Day 4, MAb-9D5 treated mice had mild ISH labeling corresponding with to areas of necrosis/inflammation. Day 8 animals had moderate lymphoid depletion and apoptosis/necrosis, indicated by areas of pallor in the white pulp, especially in the marginal zone, imparting a "moth eaten" appearance to the white pulp; ISH labeling was minimal. mAb-9D5 day 12 spleens showed mild lymphoid depletion in the white pulp with foci of inflammation and necrosis in the red pulp (and/or at the junction with the white pulp), and mild ISH labeling. Isotype controls spleens had marked lymphoid depletion with apoptosis/necrosis and hemorrhage in the white pulp, and inflammation in the red pulp. There were also marked ISH labeling. ISH stained spleens were counterstained with hematoxylin. Day 4 N=5 mice/group, day 8 N=5 mice mAb 9D5 treated, day 12 N=2 mAb 9D5 treated were evaluated and representative images depicting the most severe pathology are shown.

Table S1. Mab-9D5 does not neutralize CCHFV

	Virus strain	Antibody	PRNT50
No Complement	Afg09	9D5	<20
		11E7	>640
		6D8	<20
	IbAr 10200	9D5	<20
		11E7	>640
		6D8	<20
Complement (5%)	Afg09	9D5	<20
		11E7	>640
		6D8	<20
	IbAr 10200	9D5	<20
		11E7	>640
		6D8	<20

Table S1. Plaque reduction neutralization test (PRNT) with mAb-9D5. A dilution series from 1:20 to 1:640 of mAb-9D5, or the positive control mAb-11E7, or the negative control mAb-6D8 (anti-Ebola GP antibody) were incubated with either CCHFV strain Afg09 or IbAr 10200, with or without the addition of human complement (5% final concentration). A 50% reduction in plaques was calculated from the mAb-6D8 negative control. This experiment was conducted in duplicate.

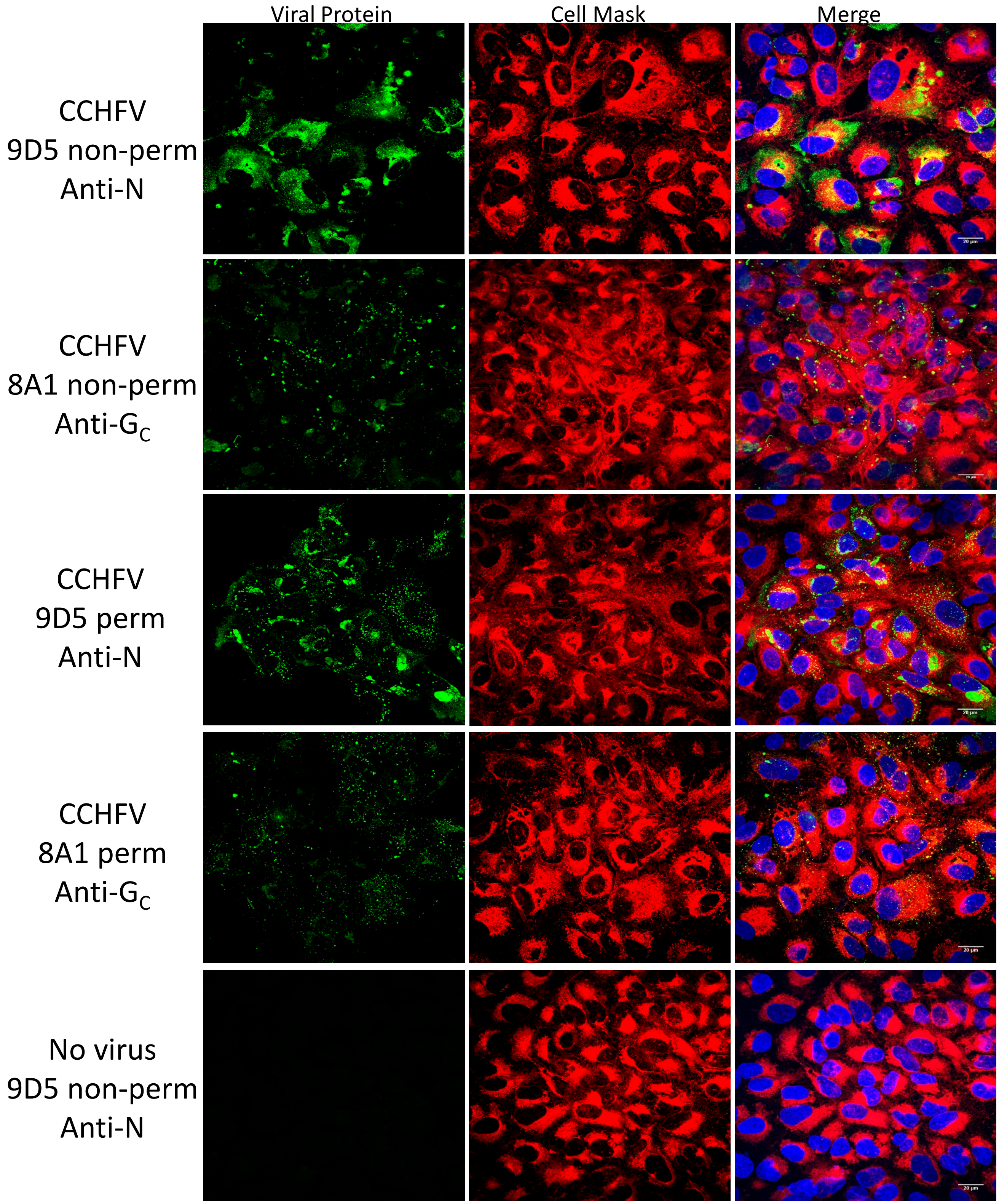

Figure S3. Surface localization of NP in CCHFV infected cells. Permeabilized and non-permeabilized A549 cells infected with CCHFV strain IbAr10200 were stained with the indicated antibodies against CCHFV viral proteins (NP;MAb-9D5 or Gc; mAb-8A1; green) and cell mask (red). Cell nuclei were stained with DAPI (blue). Two experiments were conducted with six replicates per experiment.

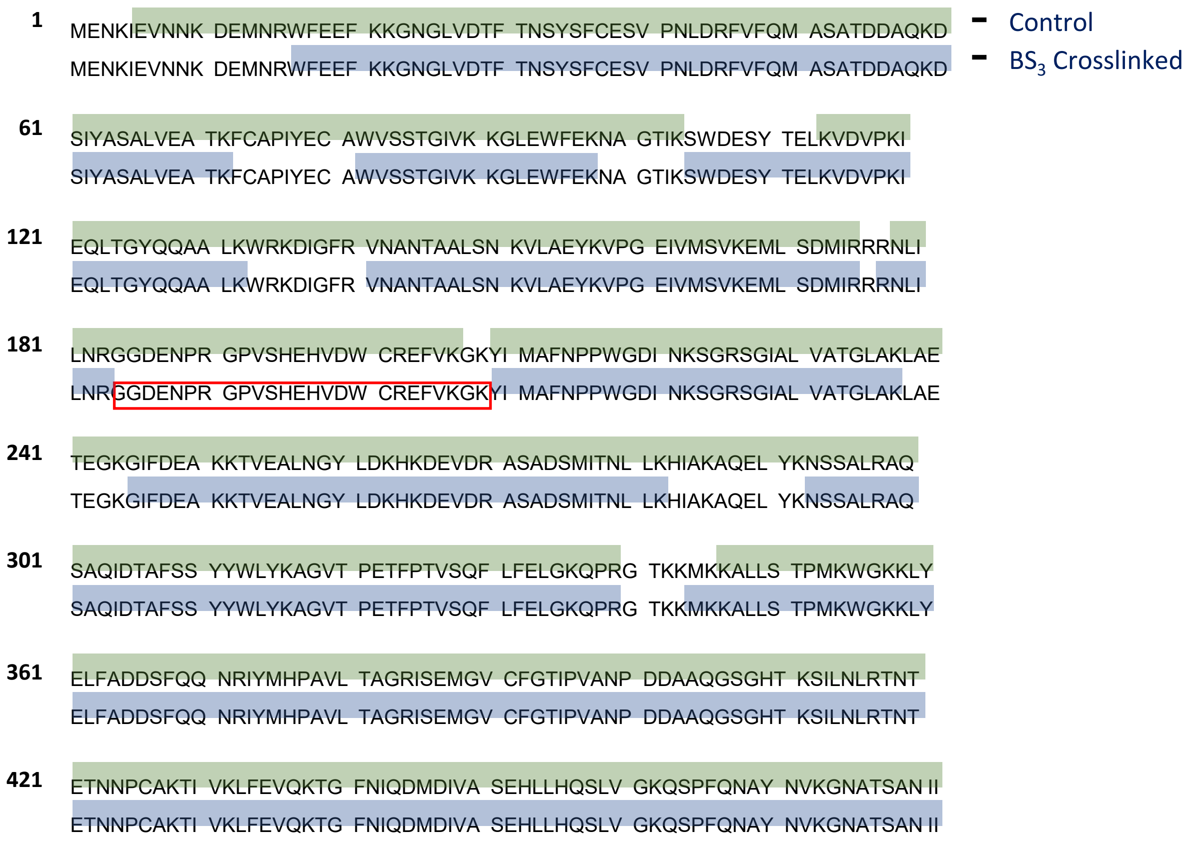

Figure S4. LC-MS epitope mapping. mAb-9D5 was bound to magnetic beads and the recombinant NP was bound to the antibody, cross-linked, and then digested with trypsin/Lyc C. As a control, a non-cross-linked sample was also analyzed. This analysis identified the mAb-9D5 protected region between amino acids 184 to 208 of IbAr 10200 N protein (Accession # MH483987.1). Red box indicates the mAb-95D binding domain. Two biological replicates were analyzed in triplicate to identify the mAb-9D5 epitope.

Percent Identical

Strain	10200	Afg09	Aigai	Erve	Hazara	Hoti	Oman	Semnya	Senegal
10200		**92.0**	**84.0**	**46.2**	**65.4**	**92.0**	**88.0**	**92.0**	**88.0**
Afg09	**92.0**		**84.0**	**46.2**	**65.4**	**100.0**	**96.0**	**100.0**	**92.0**
Aigai	**84.0**	**84.0**		**38.5**	**65.4**	**84.0**	**88.0**	**84.0**	**88.0**
Erve	**46.2**	**46.2**	**38.5**		**38.5**	**46.2**	**46.2**	**46.2**	**42.3**
Hazara	**65.4**	**65.4**	**65.4**	**38.5**		**65.4**	**65.4**	**65.4**	**61.5**
Hoti	**92.0**	**100.0**	**84.0**	**46.2**	**65.4**		**96.0**	**100.0**	**92.0**
Oman	**88.0**	**96.0**	**88.0**	**46.2**	**65.4**	**96.0**		**96.0**	**88.0**
Semnya	**92.0**	**100.0**	**84.0**	**46.2**	**65.4**	**100.0**	**96.0**		**92.0**
Senegal	**88.0**	**92.0**	**88.0**	**42.3**	**61.5**	**92.0**	**88.0**	**92.0**	

Percent Identical

Table S2. Percent similarity of the MAb-9D5 epitope. Percent amino acid identify and dissimilarity of the MAb-9D5 epitope was calculated by Geneious Prime 2023.2.

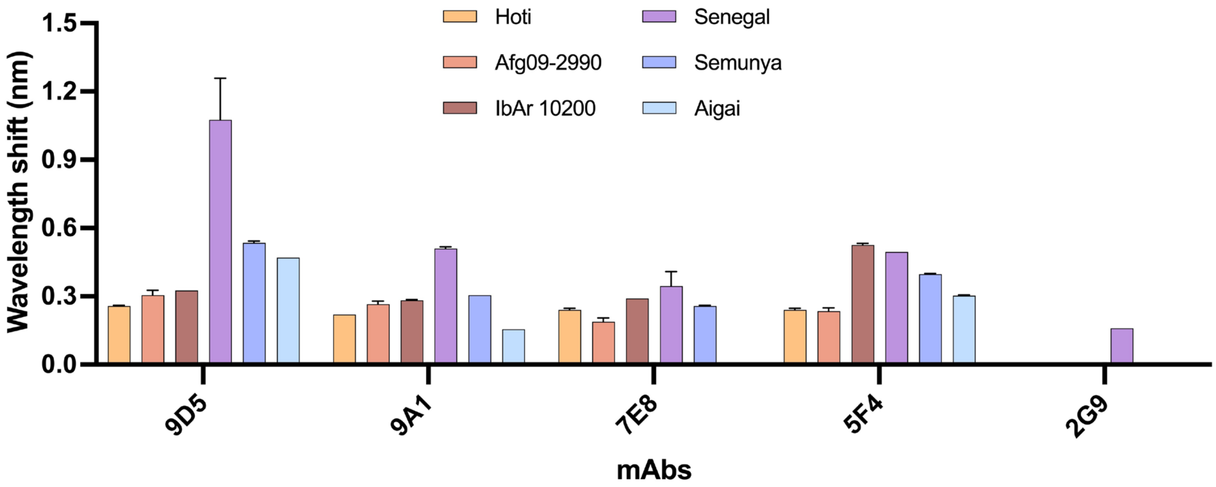

Figure S5. Mouse ascite-mAbs binding affinity screening to NP. BLI SA biosensors were loaded with NP (antigen) at concentrations of 500 nM and a single dilution (1:64) of the ascite-mAbs was employed in triplicate. Wavelength shift (nm) correspond to a semi-quantitative measurement of the binding affinities between the mAbs and NP. Erve-virus did not bind to any of the tested mAbs.

	Afg09		Hoti		IbAr 10200		Senegal		Semunya		Aigai	
**mAb-ascites**	R^2	nm shift ± SD	R^2	nm shift ± SD	R^2	nm shift ± SD	R^2	nm shift ± SD	R^2	nm shift ± SD	R^2	nm shift ± SD
**9D5**	0.99	0.305 ± 0.02	0.974	0.257 ± 0.03	0.984	0.325	0.981	1.075 ±0.183	0.985	0.535 ± 0.007	0.972	0.47
**9A1**	0.982	0.265 ± 0.014	0.99	0.22	0.982	0.2825 ± 0.003	0.98	0.51 ± 0.007	0.984	0.305	0.981	0.155
**7E8**	0.967	0.18 ± 0.017	0.973	0.24 ± 0.007	0.975	0.29	0.977	0.35 ± 0.063	0.972	0.257 ± 0.003		NB
**5F4**	0.961	0.235 ± 0.014	0.955	0.24 ± 0.007	0.968	0.525 ± 0.007	0.975	0.495	0.931	0.397 ± 0.003	0.971	0.305 ± 0.003
**2G9**		NB		NB		NB	0.961	0.16		NB		NB

NP 500nM; mouse-ascites mAbs 1:64 dilution
NB: no binding
Erve virus: no binding for all tested mAbs

Table S3. Mouse ascites-mAbs to NP BLI. Wavelength shift (nm) (mean ± SD) correspond to a semi-quantitative measurement of the binding affinities between the mAbs and NP. Erve-virus did not bind to any of the tested mAbs. Samples were evaluated in triplicate.
